# Tenascin-C Drives Inflammatory VSMC Phenotypic Switching Through NF-κB Signaling in Saphenous Vein Graft Restenosis

**DOI:** 10.3390/ijms27125516

**Published:** 2026-06-18

**Authors:** Lipeng Jiang, Hongyu Gao, Tianxiang Gu, Enyi Shi

**Affiliations:** Department of Cardiac Surgery, The First Hospital of China Medical University, No. 155 Nanjing North Street, Shenyang 110001, China; 2023121076@cmu.edu.cn (L.J.); 2023120791@cmu.edu.cn (H.G.)

**Keywords:** vein graft restenosis, tenascin-C, vascular smooth muscle cell, maladaptive remodeling, inflammation, NF-κB signaling

## Abstract

Vein graft restenosis is a leading cause of long-term failure after coronary artery bypass grafting (CABG), driven by maladaptive vascular smooth muscle cell (VSMC) responses to arterialization-induced inflammation. The key molecular mediators of this pathological remodeling, however, remain incompletely defined. Here, we integrated multi-omics analyses of human and canine vein graft specimens with in vitro functional assays to identify tenascin-C (TNC)—a matricellular extracellular matrix protein—as a critical regulator of VSMC dysfunction. TNC was specifically enriched in a synthetic, pro-inflammatory VSMC subpopulation. Pro-inflammatory stimuli potently induced TNC expression, which was functionally linked to VSMC phenotypic modulation, hyperproliferation, and enhanced migration. Mechanistically, TNC acts upstream of NF-κB signaling; siRNA-mediated TNC knockdown significantly reduced nuclear p65 protein levels and attenuated inflammatory responses. Our integrated computational and experimental data suggest that TNC, NF-κB, and TNF-α function within a sequential pro-inflammatory signaling cascade that sustains vascular inflammation and promotes neointimal hyperplasia. These findings reposition TNC from a passive structural component to an active driver of vascular pathology and highlight the TNC–NF-κB axis as a candidate target for therapeutic intervention to improve vein graft patency.

## 1. Introduction

Coronary artery bypass grafting (CABG) is a cornerstone therapy for complex coronary artery disease, with saphenous vein grafts (SVGs) being the most commonly used vascular conduit. Despite its widespread use, the long-term efficacy of this procedure is significantly limited by progressive SVG restenosis, resulting in a 10-year patency rate of only approximately 50% [1]. This luminal narrowing is primarily driven by neointimal hyperplasia, constituting a major clinical bottleneck that directly impacts patient prognosis and increases the need for repeat revascularization [2].

The fundamental cause of restenosis after vein grafting lies in the maladaptive remodeling of the venous graft in response to the arterial hemodynamic environment. This process is initiated by surgical trauma and the ensuing acute inflammatory response, coupled with dramatic alterations in flow patterns. Unlike the laminar flow in the venous system, the arterialized vein is exposed to higher pressure and shear stress, particularly at anastomotic sites and areas of curvature where disturbed flow and low-magnitude oscillatory shear stress (OSS) readily develop [3]. This abnormal mechanical environment impairs endothelial function, downregulates nitric oxide (NO) production, and activates multiple intracellular signal transduction pathways, ultimately leading to the pathological activation of VSMCs [4].

This activation is characterized by a pathological phenotypic switch of VSMCs from a quiescent, contractile state to a proliferative, migratory, and synthetic phenotype. This process is exquisitely regulated by the precise coordination of mechanical forces and biochemical signaling networks. For instance, the mTORC2 signaling pathway plays a pivotal role in vascular remodeling following vein grafting, with its activation closely associated with the upregulation of downstream protein kinase C (PKC) expression [5]. Concurrently, mechanical stress has been shown to directly activate the mechanosensitive PKC isoform PKCδ, thereby modulating vascular smooth muscle cell migration [6]. Together, these findings suggest that mTORC2 and mechanosensitive PKC isoforms may act in concert to drive pathological smooth muscle cell activation during venous arterialization. Furthermore, the mechanosensitive ion channel PIEZO1 plays a critical role in VSMC phenotypic switching and neointima formation: VSMC-specific knockout of PIEZO1 significantly suppresses intimal hyperplasia in both carotid stenosis and guidewire injury models; clinical samples also show that PIEZO1 expression levels in the neointima are far higher than in the media [7,8]. Collectively, this evidence indicates that the interplay between mechanosensation (e.g., via PIEZO1) and downstream signaling pathways forms the key molecular basis driving restenosis.

However, among the numerous regulatory factors, the key molecular integrator that couples critical inflammatory signals with dynamic extracellular matrix (ECM) changes remains poorly defined. The ECM is not a passive scaffold but an active signaling platform; its dysregulation during vein graft adaptation creates a microenvironment that supports sustained VSMC activation and chronic inflammation. Among ECM components, matricellular proteins—transiently expressed during tissue stress—have emerged as critical regulators of cell–matrix crosstalk [9]. TNC, a prototypical stress-induced matricellular glycoprotein, is strongly upregulated in various inflammatory and fibrotic diseases [10,11]. Yet, its role in SVG restenosis has not been explored. Given that TNC has been shown to regulate inflammation and cellular plasticity, we hypothesize that it may serve as a crucial nexus linking the inflammatory microenvironment to VSMC phenotypic regulation.

Notably, the NF-κB pathway is a central transcriptional regulator of inflammatory responses, controlling the expression of cytokines, adhesion molecules, and growth factors that drive VSMC proliferation and migration [12]. In non-vascular diseases (such as rheumatoid arthritis and post-myocardial infarction cardiac remodeling), studies have shown that TNC can activate NF-κB signaling through receptors like TLR4 or integrins, thereby amplifying pro-inflammatory cascades [13]. However, does the TNC–NF-κB axis play a role in vein graft lesions? If so, how does it sustain the self-perpetuating inflammatory process underlying restenosis? These questions remain entirely unknown.

Here, by integrating multi-omics analyses of human and canine vein graft specimens with rigorous in vitro functional validation, we establish TNC as a central pathogenic driver of SVG restenosis. We demonstrate that TNC is specifically enriched in a pro-inflammatory, synthetic VSMC subpopulation and is indispensable for key pathological processes, including phenotypic switching, excessive proliferation, and enhanced migratory capacity. Mechanistically, TNC acts upstream of the NF-κB signaling pathway and is closely associated with its activation, driving vascular inflammation and neointimal hyperplasia.

In summary, our study redefines TNC from a passive structural matrix component to an active regulator of vascular remodeling, highlighting its pivotal role as a nodal regulatory factor within the TNC–NF-κB axis and providing a novel molecular foundation and therapeutic target for precise intervention in SVG restenosis.

## 2. Results

### 2.1. Tenascin-C Governs the Synthetic Phenotypic Switch of Vascular Smooth Muscle Cells to Drive Vein Graft Restenosis

**Figure 1:**
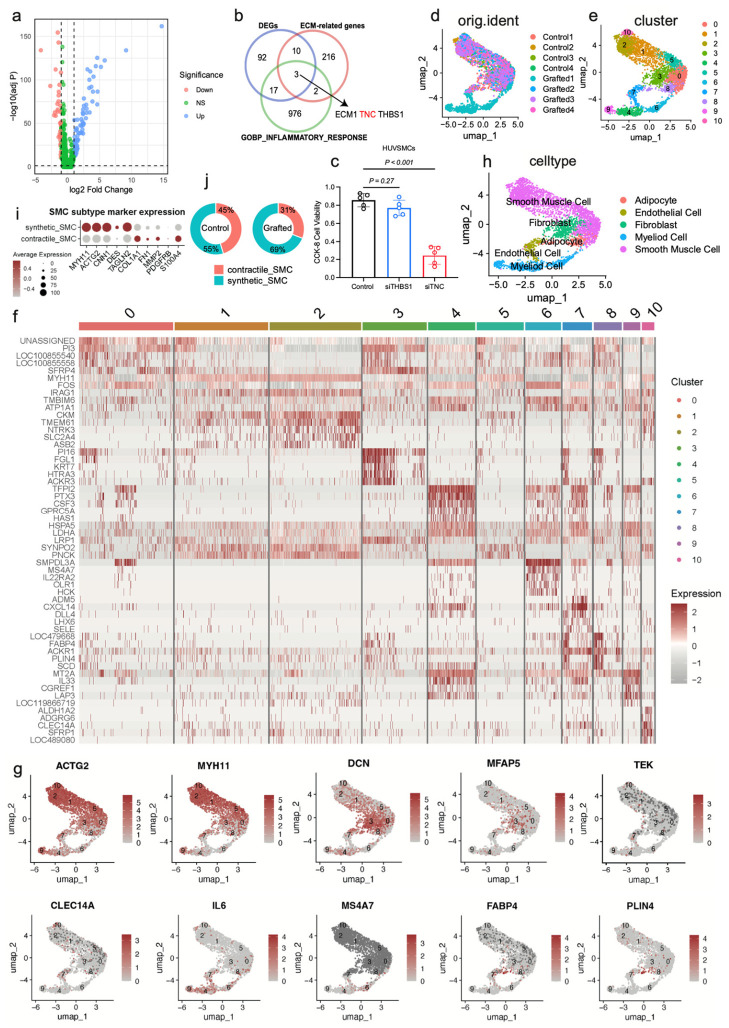
Integrated bioinformatic and functional screening identifies TNC as a key driver of vein graft restenosis. (**a**) Volcano plot depicting differentially expressed genes (DEGs) in stenotic versus patent human vein grafts from the GSE241205 dataset. Significantly upregulated (red, *n* = 83) and downregulated (blue, *n* = 39) genes are defined by |log_2_(fold change)| > 1 and adjusted *p* < 0.05. Vertical dashed lines: log_2_(fold change) = ±1; horizontal dashed line: −log_10_(adj. *p*) = 1.301 (adj. *p* = 0.05). (**b**) Venn diagram illustrating the intersection of three gene sets: 122 restenosis-associated DEGs, ECM-related genes (NABA_ECM_GLYCOPROTEINS), and inflammation/injury-related genes (GO:0006954). The core intersecting genes (ECM1, THBS1, TNC) are highlighted. (**c**) CCK-8 proliferation assay in human umbilical vein smooth muscle cells (HUVSMCs) following siRNA-mediated knockdown of THBS1 or TNC. Data are mean ± SEM; *n* = 5 biological replicates per group; *p* < 0.001 vs. si-NC and si-THBS1 groups by one-way ANOVA with Tukey’s post hoc test. (**d**) UMAP visualization of single-cell RNA sequencing (scRNA-seq) data (GSE263281) from canine vein grafts at 24 h post-surgery, showing 11 transcriptionally distinct cell clusters. (**e**) UMAP plot from (**d**) colored by experimental group (Control: Control1–Control4; Grafted: Grafted1–Grafted4), revealing altered cellular composition and distribution after arteriovenous grafting. (**f**) Heatmap of the top 5 differentially expressed genes for each of the 11 clusters identified in (**d**). Gene expression is row-scaled (Z-score). (**g**) UMAP plots displaying the expression of canonical lineage marker genes (ACTG2, PECAM1, PDGFRA, CD68, ADIPOR1) used for cell type annotation. (**h**) Bar graph quantifying the proportion of annotated cell types (SMCs, ECs, Fibroblasts, Myeloid cells, Adipocytes) in control and grafted veins. SMCs are significantly expanded in grafted samples. (**i**) Dot plot showing mutually exclusive expression of contractile (MYH11, ACTG2, CNN1, DES, TAGLN) and synthetic (COL1A1, FN1, MMP2, PDGFRB, S100A4) SMC markers across SMC subclusters. (**j**) Quantification of SMC phenotypic shift. The proportion of synthetic SMCs increased from 55% in controls to 69% in grafted veins, while contractile SMCs decreased correspondingly.

To identify key molecular determinants underlying human vein graft restenosis, we first performed an integrative bioinformatic analysis of the bulk RNA sequencing dataset GSE241205, which includes tissue samples from both stenotic and patent human vein grafts. Differential expression analysis using the limma package (v3.58.0) in R identified 122 significantly dysregulated genes in restenotic graft segments—83 upregulated and 39 downregulated (Figure 1a). To prioritize candidate regulators mechanistically linking extracellular matrix (ECM) remodeling and inflammatory responses, we intersected three gene sets: (i) the aforementioned 122 restenosis-associated differentially expressed genes (DEGs); (ii) ECM-related genes from the NABA_ECM_GLYCOPROTEINS database; and (iii) injury- and inflammation-associated genes annotated under Gene Ontology term GO:0006954. This stringent integrative approach yielded three high-confidence candidates: ECM1, THBS1, and TNC (Figure 1b).

We next conducted functional validation in human umbilical vein smooth muscle cells (HUVSMCs). Among these candidates, ECM1 encodes a structural ECM component; although significantly differentially expressed, it is not typically considered a signaling or regulatory molecule. We therefore focused on THBS1 and TNC. siRNA-mediated knockdown coupled with CCK-8 proliferation assays revealed that only TNC silencing (si-TNC) significantly suppressed HUVSMC proliferation, whereas THBS1 knockdown showed no discernible effect (Figure 1c). Consequently, we selected TNC for in-depth mechanistic investigation.

Given that pathological vascular remodeling in vein grafts is characterized by a phenotypic switch of VSMCs from a quiescent, contractile state to a proliferative, synthetic phenotype, we hypothesized that TNC may drive this transition. To test this, we leveraged a publicly available single-cell RNA sequencing (scRNA-seq) dataset (GSE263281) derived from a canine external jugular vein–carotid artery interposition graft model at 24 h post-surgery. After stringent quality control (nFeature_RNA: 200–6000; mitochondrial gene content < 10%) and cell cycle regression using Seurat (v5.0.2), unsupervised clustering resolved 11 transcriptionally distinct cell clusters (Figure 1d). To assess how experimental conditions influenced cellular composition, we colored the UMAP embedding by original sample identity (orig.ident). Although partial overlap was observed between control (Control1–Control4) and grafted (Grafted1–Grafted4) samples, marked differences in cluster proportions and spatial distribution were evident (Figure 1e), indicating substantial transcriptional and compositional shifts 24 h after arteriovenous grafting.

**Figure 2:**
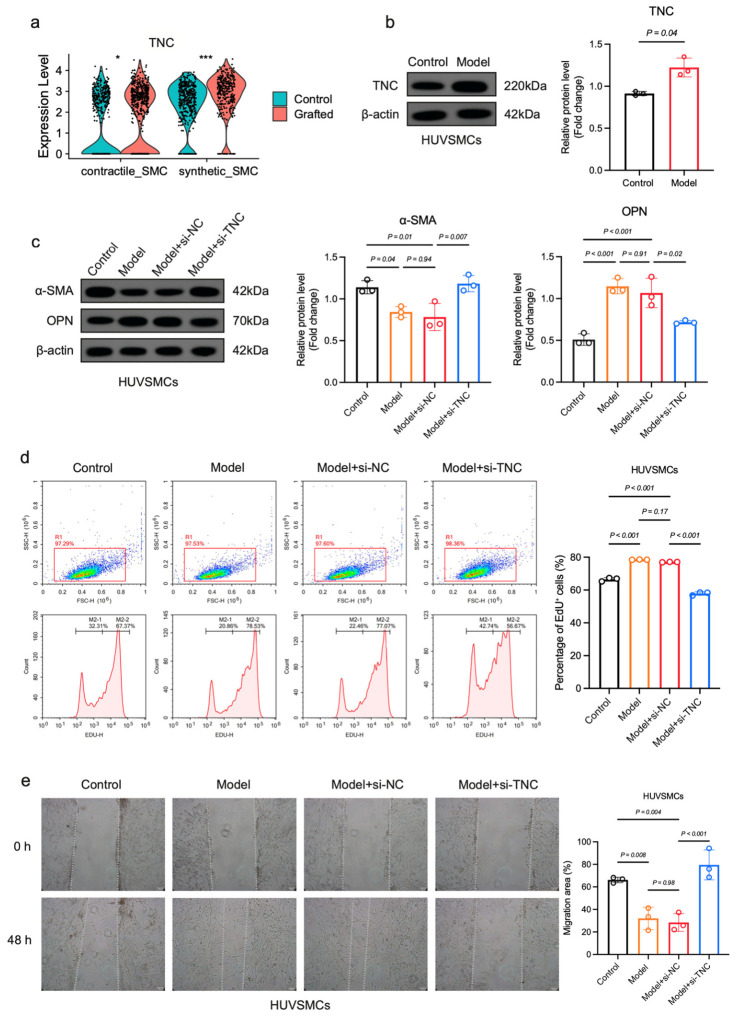
TNC is essential for inflammatory cue-induced VSMC phenotypic switching, proliferation, and migration. (**a**) Violin plots showing TNC expression levels in contractile and synthetic SMC subpopulations from control and grafted veins. TNC is significantly upregulated in both subsets post-grafting, with predominant enrichment in synthetic SMCs. *p* values were calculated using the Wilcoxon rank-sum test, *: *p* < 0.05, ***: *p* < 0.001. (**b**) Western blot analysis and quantification of TNC protein in HUVSMCs under four conditions: Control (untreated), Model (TNF-α + fibronectin), Model + si-NC, and Model + si-TNC. TNF-α/fibronectin stimulation significantly induced TNC expression. Data are mean ± SD; *n* = 3 biological replicates; *p* = 0.04 by unpaired two-tailed *t*-test. (**c**) Western blot analysis of contractile (α-SMA) and synthetic (OPN) markers in HUVSMCs under the same four conditions as in (**a**). TNC knockdown (si-TNC) reversed the model-induced loss of α-SMA and gain of OPN. Data are mean ± SD; *n* = 3 biological replicates; *p* < 0.01 vs. Model + si-NC group by one-way ANOVA with Dunnett’s post hoc test. (**d**) EdU incorporation assay to assess VSMC proliferation. Top: Representative flow cytometry plots. Bottom: Quantification of EdU^+^ cells. TNF-α/fibronectin stimulation significantly increased DNA synthesis, which was potently suppressed by TNC knockdown. Data are mean ± SD; *n* = 3 biological replicates; *p* < 0.001 by Kruskal–Wallis test with Dunn’s post hoc test. (**e**) Scratch wound healing assay to evaluate VSMC migration. Top: Representative images at 0 h and 48 h. Bottom: Quantification of the residual gap area (%). TNC knockdown significantly impaired the accelerated migration induced by the inflammatory model. Data are mean ± SD; *n* = 3 fields per group; *p* < 0.001 vs. Model + si-NC by one-way ANOVA.

Cell type annotation was performed based on cluster-specific DEGs and established vascular lineage markers. Heatmap analysis of the top five DEGs per cluster revealed highly specific marker genes (Figure 1f), which, together with their UMAP expression patterns (Figure 1g), enabled precise classification into five major vascular lineages: smooth muscle cells (SMCs), endothelial cells (ECs), fibroblasts, myeloid cells, and adipocytes. Notably, SMCs were markedly expanded in grafted veins (Figure 1h). To refine SMC phenotypes, we reclustered the SMC population using canonical markers: contractile SMCs (MYH11, ACTG2, CNN1, DES, TAGLN) and synthetic SMCs (COL1A1, FN1, MMP2, PDGFRB, S100A4). Dot plot analysis confirmed mutually exclusive expression of these marker sets (Figure 1i). Quantitative comparison revealed that the proportion of synthetic SMCs increased significantly from 55% in controls to 69% in grafted samples, concomitant with a reduction in contractile SMCs (Figure 1j), confirming arteriovenous grafting induces a pathological synthetic shift. Strikingly, TNC expression was markedly upregulated in SMCs post-grafting. Subpopulation analysis further demonstrated that TNC was predominantly enriched in synthetic SMCs, with significant elevation observed in both contractile and synthetic subsets in grafted versus control veins (Figure 2a). Collectively, these findings indicate that TNC is robustly induced following vein arteriovenous grafting, preferentially localizes to the synthetic SMC subpopulation, and may serve as a key molecular signature of this pathological phenotype during early graft injury.

To directly assess whether TNC regulates this phenotypic switch, we established an in vitro inflammatory microenvironment mimicking early graft injury by treating HUVSMCs with 10 ng/mL human TNF-α on fibronectin-coated (10 μg/mL) plates. Four experimental groups were included: (1) control (untreated); (2) model (TNF-α + fibronectin); (3) model + si-NC (scrambled siRNA control); and (4) model + si-TNC (TNC-targeting siRNA).

Western blot analysis confirmed that TNC protein expression was significantly elevated in the model group compared to control (Figure 2b), demonstrating that TNF-α potently induces TNC in VSMCs under pro-inflammatory conditions.

Subsequent Western blotting for lineage-specific markers showed that, relative to control, both the model and Model + si-NC groups exhibited markedly reduced levels of the contractile marker α-SMA and elevated levels of the synthetic marker osteopontin (OPN). Critically, TNC knockdown reversed this trend: α-SMA expression was significantly restored, while OPN was markedly suppressed in the Model + si-TNC group compared to Model + si-NC (Figure 2c). These data collectively demonstrate that TNC is essential for inflammation-driven phenotypic switching of VSMCs toward a synthetic state.

Moreover, TNC potently regulated VSMC proliferative capacity. EdU incorporation assays confirmed that TNF-α/fibronectin stimulation significantly enhanced DNA synthesis (78.53% EdU^+^ cells vs. 67.37% in control). This proliferative burst was robustly attenuated by TNC knockdown (56.67% EdU^+^ in Model + si-TNC vs. 77.07% in Model + si-NC) (Figure 2d).

Finally, scratch wound healing assays demonstrated that TNC also governs VSMC migratory potential. At 48 h, the model group exhibited markedly accelerated closure (~22% remaining gap) compared to control (~68%). si-NC had no effect, whereas si-TNC treatment significantly impaired migration, increasing the residual gap to ~80% (Figure 2e).

Collectively, our findings identify TNC as a key molecular feature of the synthetic SMC phenotype in early vein graft injury and demonstrate that TNC contributes to inflammation-induced VSMC phenotypic modulation, proliferation, and migration in vitro. These results support a pathogenic role for TNC in the early phase of vein graft remodeling and suggest it may represent a candidate target for further investigation.

### 2.2. Tenascin-C Promotes Pathological Remodeling of Vein Grafts via NF-κB–Dependent Inflammatory Signaling

**Figure 3:**
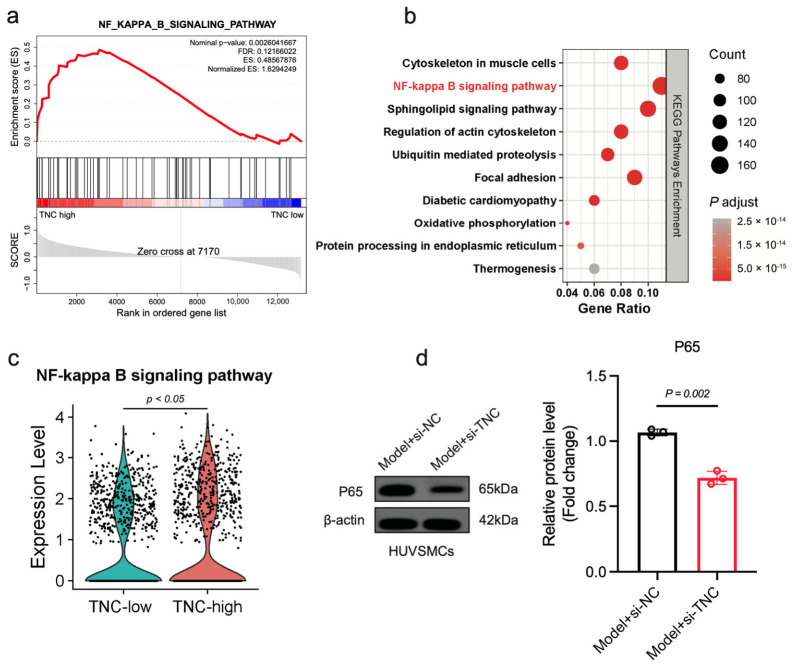
TNC is enriched in and functionally required for NF-κB pathway activation in venous SMCs. (**a**) Kinase Set Enrichment Analysis (KSEA) identifies the NF-κB signaling pathway as the top enriched KEGG pathway associated with TNC expression (Enrichment Score = 1.62; nominal *p* = 0.0026; FDR = 0.12; normalized ES = 1.624). The enrichment plot shows the running enrichment score (ES) across the ranked gene list (from TNC-low to TNC-high), with the leading-edge subset indicated by the red bar. (**b**) Bubble plot of KEGG pathway enrichment analysis, with size representing gene count and color indicating adjusted *p*-value. The “NF-kappa B signaling pathway” ranks highest among muscle cell–related pathways. (**c**) Violin plots showing significantly higher expression of NF-κB signaling pathway genes in TNC-high versus TNC-low SMC subpopulations (*p* < 0.05, Wilcoxon rank-sum test). (**d**) Western blot and quantification of nuclear p65 protein levels in VSMCs following siRNA-mediated TNC knockdown (si-TNC) or scrambled control (si-NC) under inflammatory stimulation (Model). β-actin serves as a loading control. Relative p65 protein level is normalized to β-actin and expressed as fold change relative to Model + si-NC (mean ± SEM; *n* = 3 independent experiments; *p* = 0.002, two-tailed unpaired *t*-test).

To further explore the potential molecular mechanisms by which TNC contributes to vein graft restenosis, we performed Kinase Set Enrichment Analysis (KSEA) with TNC as the central node. This analysis revealed significant enrichment of TNC within the NF-κB signaling pathway (Enrichment Score = 1.62; adjusted *p* = 0.002) (Figure 3a), suggesting a close association between TNC and this inflammatory pathway and prompting us to focus on the “TNC–NF-κB” axis in subsequent analyses.

Building on this observation, we examined NF-κB–related gene expression in TNC-high SMCs subpopulations. Using Seurat, we calculated the average expression of canonical NF-κB signature genes—including RELA, NFKB1, and IL6—as a surrogate indicator of pathway-related activity across SMC subsets. Co-expression analysis demonstrated that NF-κB–associated genes were most prominently enriched in TNC-high SMCs (Figure 3b), with significantly higher average expression compared to TNC-low counterparts (Figure 3c). Collectively, these findings indicate that TNC expression is markedly upregulated in SMCs within 24 h after arteriovenous grafting, and that TNC-high SMC subpopulations exhibit enhanced expression of NF-κB–related genes, suggesting a potential role for TNC in modulating SMC phenotypic switching during vascular remodeling following venous arterialization.

To preliminarily assess whether TNC may act upstream of NF-κB signaling, we evaluated nuclear translocation of p65—the canonical NF-κB subunit and a key component of the transcriptionally active complex—following TNC knockdown. Western blot analysis of nuclear fractions showed that siRNA-mediated silencing of TNC (Model + si-TNC) significantly reduced nuclear p65 protein levels compared to the scrambled siRNA control (Model + si-NC) (Figure 3d). Given that nuclear accumulation of p65 is commonly associated with activation of the canonical NF-κB pathway, these results suggest that TNC may contribute to the regulation of p65 nuclear localization under inflammatory conditions.

In summary, integrated computational and experimental analyses demonstrate that vein grafting rapidly induces a phenotypic shift in vascular SMCs toward a synthetic state, accompanied by robust upregulation of TNC. Notably, the TNC-high SMC subpopulation exhibits heightened expression of NF-κB–associated genes. Mechanistically, pro-inflammatory stimuli such as TNF-α potently induce TNC expression in SMCs, and TNC appears to support sustained nuclear p65 levels—as evidenced by reduced nuclear p65 upon TNC knockdown. Considering that NF-κB is a well-established transcriptional regulator of multiple pro-inflammatory mediators, including TNF-α itself, our data suggest a potential interplay among TNC, NF-κB, and TNF-α. This putative inflammatory network may help sustain vascular inflammation and promote pathological features such as SMC phenotypic modulation, hyperproliferation, and enhanced migration, thereby contributing to maladaptive vascular remodeling and ultimately neointimal hyperplasia and vein graft restenosis.

## 3. Discussion

Vein graft restenosis remains a major limitation of CABG, with its pathological core being neointimal hyperplasia driven by VSMC dysfunction [14]. This complication fundamentally arises from the maladaptive response of the venous graft to the arterial hemodynamic environment, a process in which systemic inflammation and ECM remodeling are intricately intertwined. Despite decades of research, effective preventive strategies are still lacking, partly due to the unclear identity of key molecular nodes that integrate the dynamics of inflammation and ECM changes.

In this study, by integrating transcriptomic data from human restenotic grafts with functional validation, we identified TNC—a matricellular protein traditionally regarded as a structural ECM component—as a potential active regulator in the early injury phase of vein grafts. Our bioinformatic screening strategy prioritized genes concurrently involved in both ECM remodeling and inflammatory responses, leading us to select TNC as a high-confidence candidate molecule. Although ECM1 and THBS1 were also identified, only TNC knockdown significantly suppressed VSMC proliferation, suggesting its role extends beyond mere structural support. Notably, while Wentzel et al. [15] established low shear stress as a critical hemodynamic driver of neointima formation in human coronary stents, our work provides a complementary perspective by focusing on the molecular mechanisms of inflammation-driven pathology, revealing that TNC, as a stress-induced matricellular protein, may mediate the pathological VSMC response within this inflammatory milieu.

TNC is not a constituent of the quiescent vessel wall but is an “on-demand” or provisional matrix protein typically secreted by activated VSMCs or macrophages under conditions of injury or inflammation [16]. This characteristic positions it as a potential specific marker for active vascular pathology. In our in vitro model simulating early graft injury (TNF-α + fibronectin), TNC expression was highly correlated with the phenotypic switch of VSMCs from a contractile to a synthetic state. Knockdown of TNC partially reversed the downregulation of α-SMA and the upregulation of OPN, indicating that TNC promotes the inflammation-induced phenotypic transition of VSMCs from a contractile to a synthetic state. This observation is highly consistent with the findings of Kwei et al. [17,18], who, in their murine vein graft arterization model, described VSMC activation and proliferation as early as 24 h post-arterialization. Our study provides a specific molecular mechanism for this early adaptive response, identifying TNC as a key player in promoting this phenotypic transition.

At the mechanistic level, we found a close association between TNC and the NF-κB pathway. Kinase enrichment analysis, single-cell co-expression profiling, and nuclear p65 translocation assays collectively demonstrated that, under inflammatory stimulation, the presence of TNC correlates with enhanced NF-κB pathway activity. TNC knockdown significantly reduced nuclear p65 protein levels, suggesting that TNC may be involved in regulating the nuclear translocation of p65. Although our data did not directly demonstrate a transcriptional feedback loop of NF-κB on TNF-α or TNC, given that NF-κB is a master transcriptional regulator of numerous pro-inflammatory cytokines (including TNF-α), a mutually reinforcing interaction among TNC, NF-κB, and TNF-α is plausible. This potential inflammatory regulatory network could account for the progressive nature of neointimal hyperplasia. The study by Mavromatis et al. [19] also provides relevant theoretical support; they demonstrated in an ex vivo human saphenous vein perfusion model that arterial flow conditions rapidly induce oxidative stress and MMP activation, processes known to be regulated by the NF-κB pathway. Thus, TNC may serve as a crucial amplifier of this inflammatory cascade.

This study has limitations. The HUVSMCs used, although widely employed, may not fully represent VSMCs derived from the great saphenous vein. Furthermore, the scRNA-seq data from our canine model reflect only the acute phase changes at 24 h post-surgery, lacking longitudinal dynamic information. Nevertheless, the consistency between our multi-omics and in vitro experimental results provides a solid foundation for future investigations.

In summary, our study identifies TNC as a critical factor associated with VSMC dysfunction in the early inflammatory microenvironment of vein graft restenosis. TNC is not only co-expressed with the synthetic phenotype but also promotes the pathological behavior of VSMCs in vitro. These findings support the view of TNC as a functional matrix signaling molecule in vascular remodeling and suggest that the TNC–NF-κB axis warrants further validation as a potential therapeutic target. Our work, by placing TNC within the context of established research frameworks on early VSMC phenotypic transition (Kwei) and inflammatory signaling (Mavromatis), not only corroborates prior observations but also provides deeper mechanistic insight into the molecular hub driving this complex pathological process.

## 4. Materials and Methods

### 4.1. Single-Cell RNA Sequencing Dataset Analysis

#### 4.1.1. GEO Dataset Acquisition and Experimental Model

The single-cell RNA sequencing (scRNA-seq) dataset GSE241205 was retrieved from the Gene Expression Omnibus (GEO) database (https://www.ncbi.nlm.nih.gov/geo/, accessed on 17 January 2024). This dataset comprises samples from four canines (three males, one female), with four biological replicates each from a jugular vein graft group and a matched control group. A canine jugular vein–to–carotid artery bypass grafting model was employed to recapitulate the pathophysiological cascade of human autologous vein graft injury. In this model, intraoperative venous dilation was combined with post-implantation tissue harvesting to faithfully mimic the mechanical stress (e.g., distension, handling) and biological perturbations (e.g., ischemia–reperfusion, inflammatory activation) encountered by clinical vein grafts during procurement, surgical manipulation, and engraftment. The experimental design was specifically optimized to capture both spatial heterogeneity—reflecting the layered architecture of the vascular wall—and temporal dynamics spanning the transition from acute mechanical injury to chronic neointimal remodeling.

#### 4.1.2. Quality Control, Normalization, Dimensionality Reduction, and Clustering

All computational analyses were performed in R (v4.3.1) using the Seurat package (v5.0.2; Satija Lab, New York Genome Center, New York, NY, USA) [20]. Raw count matrices were imported into the Seurat object framework. Initial quality control was conducted by calculating the percentage of mitochondrial gene expression (genes prefixed with “MT-”) and ribosomal protein gene expression (genes prefixed with “RPL” or “RPS”) per cell using the PercentageFeatureSet function. Cells exhibiting >20% mitochondrial reads or <200 detected genes were excluded as low-quality or apoptotic; cells with >6000 detected genes or >10% ribosomal reads were flagged as potential doublets and removed.

Data normalization and variance stabilization were performed using the regularized negative binomial regression method implemented in SCTransform, which simultaneously identified 2000–3000 highly variable genes (HVGs) while regressing out technical confounders [21]. Principal component analysis (PCA) was applied to the HVGs, and the top 20–30 principal components (PCs)—selected based on elbow plots and biological interpretability—were retained for downstream analysis. A shared nearest neighbor (SNN) graph was constructed using the FindNeighbors function with k = 30. Unsupervised clustering was performed via the Louvain algorithm (FindClusters) with a resolution parameter of 0.8, yielding 11 transcriptionally distinct clusters (designated Cluster 0–10). Non-linear dimensional reduction and visualization were achieved using Uniform Manifold Approximation and Projection (UMAP) via the RunUMAP function, using the same set of PCs. Cluster identities were initially assigned by identifying differentially expressed genes (DEGs) for each cluster against all others using FindAllMarkers with default parameters (log_2_ fold-change threshold ≥ 0.25, minimum percent expression ≥ 0.25).

#### 4.1.3. Cell Type Annotation

Cell types were annotated by cross-referencing cluster-specific marker genes with established lineage-defining markers from the vascular biology literature (e.g., PECAM1/CD31 for endothelial cells, ACTA2/SMA for smooth muscle cells, CD68 for macrophages, VWF for endothelial subtypes). Final annotations were validated by projecting the expression of canonical marker genes onto the UMAP embedding using the FeaturePlot function to confirm their specific enrichment within expected populations. Comprehensive visualizations of cluster identity, sample origin (graft vs. control), and annotated cell types were generated using the DimPlot function with consistent color schemes across figures.

#### 4.1.4. Cellular Composition Analysis and Smooth Muscle Cell (SMC) Subclustering

The relative abundance of each annotated cell type was quantified as the proportion of cells per sample. Group-level comparisons of cellular proportions between graft and control groups were assessed using chi-square tests for categorical frequency data or two-sample *t*-tests for continuous proportions (after arcsine-square root transformation to stabilize variance), as appropriate. Results were visualized as stacked bar plots (for relative frequencies) and grouped bar charts (for absolute cell counts) using the ggplot2 package (v3.4.0; Wickham et al. [22]).

To resolve phenotypic heterogeneity within vascular smooth muscle cells, the SMC population—defined by high expression of ACTA2 (smooth muscle actin alpha-2)—was isolated for secondary analysis. This subset underwent re-normalization using SCTransform, followed by PCA, graph-based clustering (FindNeighbors with k = 20; FindClusters with resolution = 0.6), and UMAP visualization. This refined analysis revealed two dominant SMC states: contractile (characterized by high expression of MYH11, ACTG2, CNN1, DES) and synthetic (marked by elevated COL1A1, FN1, MMP2, PDGFRB, S100A4) [23]. Subtype assignments were visualized using DimPlot with overlays for UMAP clusters, experimental group (graft vs. control), and SMC phenotype.

#### 4.1.5. Differential Gene Expression in Key Subpopulations

Differential expression analysis was performed separately for endothelial cells and SMCs comparing graft versus control conditions. For endothelial cells, the top five most significantly up- and down-regulated DEGs (ranked by adjusted *p*-value) were visualized using the scRNAtoolVis R package (v1.2.0). Within the SMC subpopulation, DEGs were identified using the Wilcoxon rank-sum test via FindMarkers, with log_2_ fold-changes (log_2_FC) and Benjamini–Hochberg-adjusted *p*-values computed for all genes. Results were displayed as volcano plots generated with ggplot2, with significance thresholds defined as |log_2_FC| ≥ 0.5 and adjusted *p*-value < 0.05.

#### 4.1.6. Gene Set Enrichment Analysis (GSEA)

To investigate the functional implications of TNC expression in SMCs, the SMC population was stratified into “TNC-high” and “TNC-low” groups based on median TNC expression across all SMCs. Genes were ranked by log_2_FC from the TNC-high versus TNC-low comparison and subjected to preranked gene set enrichment analysis (GSEA) [24] using the fgsea R package (v1.28.0; Korotkevich et al. [25], Russia). The analysis was performed against a curated collection of pathways related to vascular inflammation, extracellular matrix remodeling, and NF-κB signaling (derived from KEGG, Reactome, and Hallmark databases). Enrichment of the NF-κB signaling pathway is highlighted, with the Normalized Enrichment Score (NES), false discovery rate (FDR) q-value (<0.05 considered significant), and core enriched genes explicitly annotated in the enrichment plot.

### 4.2. Cell Culture and Transfection

Human umbilical artery smooth muscle cells (HUASMCs) were cultured in endothelial cell medium (ECM; ScienCell Research Laboratories, Carlsbad, CA, USA) supplemented with 5% fetal bovine serum (FBS) and endothelial cell growth supplement (ECGS). Cells were maintained at 37 °C in a humidified incubator with 5% CO_2_. HUASMCs at logarithmic growth phase were seeded into 6-well plates and transfected with small interfering RNAs (siRNAs) using Lipofectamine RNAiMAX Reagent (Invitrogen, Carlsbad, CA, USA) according to the manufacturer’s protocol. The following experimental groups were established: Control (C), Negative Control (NC), si-TNC-1 (Si-1), si-TNC-2 (Si-2), and si-TNC-3 (Si-3). Transfection efficiency was validated by Western blotting and quantitative real-time PCR (qRT–PCR) 48 h post-transfection.

For functional studies, HUASMCs were divided into four groups:

Control group: Cells cultured under normal conditions.

Model group: Cells stimulated with human TNF-α (10 ng/mL; PeproTech, Cranbury, NJ, USA) and plated on culture dishes pre-coated with fibronectin (10 μg/mL; Corning, NY, USA).

Model + si-NC group: Model group cells transfected with a non-targeting, scrambled siRNA (RiboBio, Guangzhou, China).

Model + si-TNC group: Model group cells transfected with TNC-targeting siRNA (RiboBio, China) to silence Tenascin-C expression.

### 4.3. qRT–PCR

Total RNA was extracted from cultured cells using TRIzon reagent (Accurate Biology, Hunan, China) following the manufacturer’s instructions. Briefly, cells were lysed directly in the culture plate by adding 1 mL of TRIzon per 10 cm^2^ of surface area and homogenized by repeated pipetting. The lysate was incubated at room temperature for 5 min. Subsequently, 0.2 mL of chloroform was added per 1 mL of TRIzon, and the mixture was vortexed vigorously for 15 s and incubated at room temperature for 2–3 min. The sample was then centrifuged at 12,000 rpm (~13,400× *g*) for 15 min at 4 °C. The upper aqueous phase was carefully transferred to a new RNase-free tube. RNA was precipitated by adding an equal volume of isopropanol, incubating at room temperature for 10 min, and centrifuging at 12,000 rpm for 10 min at 4 °C. The RNA pellet was washed with 75% ethanol, air-dried, and dissolved in RNase-free water. RNA concentration and purity were assessed by spectrophotometry (A260/A280 > 1.9).

One microgram of total RNA was reverse-transcribed into cDNA using the All-in-One qRT Master Mix (Accurate Biology) in a 20 μL reaction volume. qRT–PCR was performed in triplicate on a QuantStudio™ real-time PCR system (Thermo Fisher Scientific, Waltham, MA, USA) using SYBR Green chemistry. Each 10 μL reaction contained 5 ng of cDNA template and gene-specific primers (200 nM final concentration). Amplification conditions consisted of an initial denaturation at 95 °C for 3 min, followed by 40 cycles of 95 °C for 10 s and 60 °C for 30 s. Gene expression was normalized to β-actin and calculated relative to the control group using the ΔΔCt method.

### 4.4. Western Blot

Cells in 6-well plates were washed three times with ice-cold PBS and lysed with 300 μL of ice-cold lysis buffer (Beyotime, Shanghai, China) containing 1% protease inhibitor cocktail (MedChemExpress, Monmouth Junction, NJ, USA). The lysate was collected, incubated on ice for 15 min with agitation, and centrifuged at 12,000× *g* for 15 min at 4 °C. The supernatant containing total cellular protein was aliquoted and stored at −80 °C.

Protein concentration was determined using the BCA Protein Assay Kit (Beyotime) with BSA as the standard. Equal amounts of protein were separated by SDS-PAGE on 8–12% gels and transferred onto 0.22 μm PVDF membranes (Millipore, Burlington, MA, USA). Membranes were blocked with 5% non-fat milk in TBST for 1 h at room temperature, followed by overnight incubation at 4 °C with primary antibodies diluted according to the manufacturer’s instructions. The next day, membranes were washed three times with TBST and incubated with horseradish peroxidase (HRP)-conjugated secondary antibodies (Proteintech Group, Inc., Wuhan, China) for 1 h at room temperature. After washing, chemiluminescent signals were detected using an ECL substrate (Beyotime) and captured with a high-sensitivity chemiluminescence imaging system (Tanon, Shanghai, China). Band intensities were quantified by densitometry using ImageJ software 1.54P (NIH, Bethesda, MD, USA), and target protein levels were normalized to GAPDH or β-actin as loading controls.

### 4.5. Cell Proliferation Assay

Cell proliferation was assessed using the Cell Counting Kit-8 (CCK-8; Dojindo Laboratories, Kumamoto, Japan). Human umbilical artery smooth muscle cells (HUASMCs) were seeded into 96-well plates at a density of 1 × 10^4^ cells per well in 100 μL of complete medium and allowed to adhere overnight. Following the indicated treatments, the culture medium was replaced with fresh basal medium containing 10% (*v*/*v*) CCK-8 reagent. After incubation for 1–4 h at 37 °C, the absorbance at 450 nm was measured using a microplate reader (BioTek Instruments, Winooski, VT, USA). Each experimental condition was performed in quintuplicate. Cell viability was calculated relative to the control group using the following formula: Cell viability (%) = ×100, where ODBlank represents the absorbance of wells containing medium and CCK-8 reagent without cells.

### 4.6. EdU Incorporation Assay

Cell proliferation was assessed using the EdU (5-ethynyl-2′-deoxyuridine) incorporation assay. HUASMCs were seeded into 6-well plates and subjected to the indicated treatments. Cells were then incubated with 10 μM EdU for 2 h. Following incubation, cells were fixed with 4% paraformaldehyde for 15 min at room temperature and permeabilized with 0.3% Triton X-100 in PBS for 10 min. EdU-labeled DNA was detected via a copper-catalyzed “click” reaction with an Alexa Fluor 488 azide dye according to the manufacturer’s protocol (Beyotime Biotechnology, Shanghai, China). The fluorescence intensity of incorporated EdU was quantified by flow cytometry (BD Biosciences, Franklin Lakes, NJ, USA).

### 4.7. Wound Healing Assay

For the wound healing assay, HUASMCs were seeded into 6-well plates and cultured until they reached confluence (>90%). A sterile 200-μL pipette tip was used to create a straight scratch wound across the monolayer. The wells were then washed three times with PBS to remove detached cells and replenished with serum-free medium supplemented with 0.1% BSA. Images of the wound area were captured at 0 and 48 h post-scratching at predetermined locations using an inverted microscope (Olympus, Tokyo, Japan). The migration distance was calculated as the difference between the initial and final wound widths, measured using ImageJ software (NIH, Bethesda, MD, USA). The cell migration rate was determined using the formula: Migration rate (μm/h) = Migration distance (μm)/Migration time (h).

### 4.8. Statistical Analysis

All statistical analyses were performed using SPSS 26.0 (IBM, Armonk, NY, USA) and GraphPad Prism 9.0 (GraphPad Software, San Diego, CA, USA). Data are presented as mean ± standard deviation (SD) or median with interquartile range [M (Q1, Q3)], as appropriate. Normality was assessed using the Shapiro–Wilk test. For comparisons between two groups, an unpaired Student’s *t*-test (for normally distributed data) or a Mann–Whitney U test (for non-normally distributed data) was used. For comparisons among multiple groups, one-way analysis of variance (ANOVA) followed by Tukey’s post hoc test (for normally distributed data) or Kruskal–Wallis test followed by Dunn’s post hoc test with Bonferroni correction (for non-normally distributed data) was applied. A two-sided *p* value < 0.05 was considered statistically significant.

## 5. Conclusions

By integrating multi-omics analyses with in vitro functional assays, we demonstrate that TNC is significantly upregulated during the early phase of vein graft arterization and is preferentially enriched within the synthetic VSMC subpopulation. Functionally, TNC expression is closely associated with VSMC phenotypic switching, hyperproliferation, and enhanced migratory capacity. Mechanistically, TNC exhibits a strong correlation with NF-κB pathway activity, and its knockdown markedly reduces nuclear p65 levels, indicating a regulatory role for TNC in NF-κB signaling under inflammatory conditions. These findings redefine TNC as an active participant in vascular inflammation and remodeling, providing a preliminary rationale for targeting the TNC–NF-κB axis as a therapeutic strategy to improve long-term vein graft patency.

## Data Availability

The datasets used and/or analyzed during the current study are available from the corresponding authors on reasonable request.

## Abbreviations

The following abbreviations are used in this manuscript:

CABG	Coronary artery bypass grafting
SVGs	Saphenous vein grafts
VSMCs	Vascular smooth muscle cells
ECM	Extracellular matrix
TNC	Tenascin-C
NF-κB	Nuclear factor kappa-light-chain-enhancer of activated B cells
TNF-α	Tumor necrosis factor alpha
SMC	Smooth muscle cell
KSEA	Kinase Set Enrichment Analysis
P65	Protein 65
IL6	Interleukin 6
